# Spatial distribution and cluster analysis of sexual risk behaviors reported by young men in Kisumu, Kenya

**DOI:** 10.1186/1476-072X-9-24

**Published:** 2010-05-22

**Authors:** Nelli Westercamp, Stephen Moses, Kawango Agot, Jeckoniah O Ndinya-Achola, Corette Parker, Kevine O Amolloh, Robert C Bailey

**Affiliations:** 1 School of Public Health, University of Illinois at Chicago, 1603 W Taylor Street, (MC 923), Chicago, IL, 60612-4394, USA; 2 Department of Medical Microbiology, University of Manitoba, Winnipeg, MB, R3T 2N2, Canada; 3 UNIM Project, PO Box 1764-40100, Kisumu, Kenya; 4 Department of Medical Microbiology, University of Nairobi, PO Box 30197-00100 Nairobi, Kenya; 5 RTI International, 3040 East Cornwallis Road, Post Office Box 12194, Research Triangle Park, NC 27709-2194, USA

## Abstract

**Background:**

The well-established connection between HIV risk behavior and place of residence points to the importance of geographic clustering in the potential transmission of HIV and other sexually transmitted infections (STI).

**Methods:**

To investigate the geospatial distribution of prevalent sexually transmitted infections and sexual behaviors in a sample of 18-24 year-old sexually active men in urban and rural areas of Kisumu, Kenya, we mapped the residences of 649 men and conducted spatial cluster analysis. Spatial distribution of the study participants was assessed in terms of the demographic, behavioral, and sexual dysfunction variables, as well as laboratory diagnosed STIs. To test for the presence and location of clusters we used Kulldorff's spatial scan statistic as implemented in the Satscan program.

**Results:**

The results of this study suggest that sexual risk behaviors and STIs are evenly distributed in our sample throughout the Kisumu district. No behavioral or STI clusters were detected, except for condom use. Neither urban nor rural residence significantly impacted risk behavior or STI prevalence.

**Conclusion:**

We found no association between place of residence and sexual risk behaviors in our sample. While our results can not be generalized to other populations, the study shows that geospatial analysis can be an important tool for investigating study sample characteristics; for evaluating HIV/STI risk factors; and for development and implementation of targeted HIV and STI control programs in specifically defined populations and in areas where the underlying population dynamic is poorly understood.

## Introduction

The well-established connection between HIV risk behavior and place of residence points to the importance of geographic clustering in the potential transmission of HIV and other sexually transmitted infections (STIs) [1-3]. Rothenberg et al. hypothesized that one of the elements that plays an important role in maintaining endemicity of HIV in high prevalence settings is the geographic range of persons in a social network [1,2]. The correlation between the geographical proximity of persons connected within a network, but not necessarily known to each other [2], was found to be an important factor in maintenance of HIV endemicity due to a high probability of partner selection from within the network. Latkin et al. found an association between the characteristics of neighborhood and the prevalence of sexually transmitted infections and sexual risk behaviors [3]. However, there have been few recent studies evaluating the spatial distribution of STIs, HIV, and sexual behavior. Most studies collect and analyze spatial variables on a relatively crude scale, such as neighborhood, census track, zip code, or by classifying the participants' residences as urban or rural.

The current study represents a continuation of earlier work focusing on the identification of "high transmission areas". Such areas are locations where social mixing is combined with commercial activity, such as trading centers, truck stops, and places with high concentrations of migrants [4,5]. These high transmission locations are believed to be concentrated in urban areas where HIV seroprevalence is higher than in neighboring rural areas [6-10]. However, HIV prevalence in rural areas is not homogenous, with large differences in prevalence between different settlements [11-14]. Previous research on rural-urban differences in HIV prevalence and sexual behavior led to the following opposing predictions. Some researchers suggest that established epidemics may involve urban and rural communities interacting in a complex manner with in- and out-migration patterns leading to eventual equaling of HIV prevalence in urban and rural locations [7]. An opposing opinion, that rural HIV prevalence will always be lower than comparable urban areas, maintains that sexual behaviors in urban settings provide increased opportunity for commercial and casual sex and degrade protective cultural traditions regulating sexual relationships [15,16]. Most HIV research comparing risky sexual behaviors in rural and urban settings [7,17-19] have observed behavioral differences between these populations, with higher HIV risk behaviors more frequently found in urban areas. However, in some places in rural India, it has been shown that sexual networks are such as to maintain HIV transmission independently of urban locations, with HIV prevalence in some rural areas higher than in comparable urban locations [11,12]. A study in Kenya also found that HIV risk behavior was more prevalent among women in rural compared to urban areas of Nyanza Province [20].

The objective of our study was to investigate a geospatial dimension of the distribution of STI and sexual behaviors in a sample of 18-24 year-old sexually active men in Kisumu, Kenya. Mapping participants' residences allowed for cluster analysis based on home location. Additionally, by incorporating spatial measurements with demographic, behavioral, and STI data it was possible to assess associations between residence location, sexual behavior, and prevalence of STIs.

## Methods

### Study location

The study took place in Nyanza Province, Kisumu district (presently Kisumu East and Kisumu West districts), located in western Kenya. The study area primarily includes the Kisumu East district and covers approximately 240 km^2^. Kisumu East district consists almost entirely of the Municipality of Kisumu, the third largest city in Kenya, with a population of approximately 500,000 residents [21]. The district is located on Lake Victoria, 10 km south of the equator, and approximately 1,100 meters above sea level.

### Population and study design

In order to investigate the safety and effectiveness of male circumcision as a potential HIV prevention strategy, a randomized controlled trial (RCT) of male circumcision to reduce HIV incidence in young men was conducted in Kisumu, Kenya [22]. To explore the determinants of the socio-economic status, access to healthcare, and spatial characteristics of the trial participants, including the geographic distribution of sexually transmitted infections (STI) and risk behavior, we developed a nested sub-study by recruiting participants active in the trial between June 30, 2005 and March 13, 2006. In this paper, we present a spatial analysis of the baseline characteristics of these trial participants.

Respondents were trial participants who at their baseline RCT visits were uncircumcised, HIV-negative, aged 18-24, sexually active within the last 12 months and residing in Kisumu district (presently Kisumu East and Kisumu West districts). Consenting of participants and interviews were conducted by a trained, experienced male interviewer in the participants' language of choice (English, DhoLuo, or Kiswahili). This study received ethical approval from the institutional review boards at the University of Nairobi, the University of Illinois at Chicago, the University of Manitoba, and the Research Triangle Institute.

### Variables

All consenting participants were required to complete a single study visit during which they were asked to provide information on their household utilities and possessions, the healthcare facility they visited last, and geographic coordinates of their residences. To obtain geographic coordinates, participants located the residence where they lived at the time of their enrollment in the trial on a georeferenced satellite image, with assistance from a research assistant with extensive knowledge of Kisumu geography and trained in geographic information systems (GIS). Geographic coordinates were verified for 25% of participants by traveling to identified locations and mapping them with a Global Positioning System (GPS) device. All participants provided permission to link these data with their demographic, behavioral, sexual health and STI data.

Spatial distribution of the study participants was assessed in terms of the demographic, behavioral, sexual dysfunction and laboratory diagnosed STI variables recorded at the baseline RCT visit. Demographic variables included age, education, income, importance of religion, and marital status. Behavioral variables included sex after or while drinking any time in the past or at last sex, age at first sex, number of partners in the last 12 months, exchanging money or gifts for sex in past 6 months, sex on the same day as meeting a partner, condom use in the past 6 months and at the last sex, insertive anal sex with a woman, and preference for dry or wet sex. Education, preference for dry or wet sex, and number of partners in the last 12 months were analyzed separately as nominal or ordinal variables as appropriate. All other variables were considered dichotomous. Sexual dysfunction lasting for two or more weeks in the last six months served as an indirect indicator of potential recent history of STIs and was assessed through six questions: lack of interest in sex, inability to climax, coming to a climax too quickly, pain during intercourse, lack of pleasure in sex, and trouble achieving or maintaining erection. Laboratory diagnosis of the following STIs was obtained from the trial baseline visit data: *Herpes simplex *type 2 (HSV2), syphilis, gonorrhea, chlamydial infection and trichomoniasis. The detailed explanation of the STI testing procedures is described elsewhere [23,24].

Geo-referencing was carried out using a GPS device of 3 meters accuracy (Magellan eXplorist 100 receiver), satellite imagery and ArcGIS 9.0 software (*Environmental Systems Research Institute, Inc. Redlands, California, USA 2004*).

### Statistical analysis

To test for the presence and location of clusters we used Kulldorff's spatial scan statistic as implemented in the Satscan program [25-29]. Kulldorff's Satscan program has been widely applied in public health research [30-34], partially due to the advantage of using a simple statistic for identifying spatial clusters based on geographic coordinates. The method uses a circular window of varying radius centered at each location that moves across the map so that at any given position the window includes different sets of neighboring residences. At each position, the radius of the circular window varies repeatedly from zero up to a set maximum radius, so that the maximum size of the window does not exceed 50 percent of the total study population. This method allows the circular window to continuously vary in both location and size, thereby creating a large number of distinct potential clusters.

The detection of clusters was performed by comparing the number of cases within the window with the number expected if cases were randomly distributed in space, using a Bernoulli model. The Bernoulli model provided the advantage of conducting a cluster analysis in the absence of data on the underlying general population density by considering the study population as a combination of cases and controls represented as a 0/1 variable investigated for clustering [26,35]. The unit of space was defined by the coordinates of the households. For ordinal and nominal variables, ordinal [36] and multinomial models [37] were used. We scanned for high rate clusters (i.e., observed number of cases exceeds the expected number of cases) and low rate clusters (i.e., expected number of cases exceeds the observed number of cases) for all dichotomous, ordinal, and nominal variables.

The likelihood was maximized over all windows, identifying the most likely or primary cluster. In addition to primary clusters, the software identified multiple secondary clusters ordering them according to the likelihood ratio. In this paper, we report primary clusters for all analyzed variables, as well as secondary clusters that do not overlap with primary clusters and have p value of less than or equal to 0.1. High and low rate clusters are reported in separate tables.

The significance of the identified clusters was tested with a likelihood ratio test with p-value based on Monte Carlo simulations. For Monte Carlo inference, 9,999 replications were performed for dichotomous variables and 999 replications for ordinal or nominal variables. The null hypothesis of no clusters was rejected when the p-value was less than or equal to 0.05. The rate ratio (RR) was defined as the ratio of observed to expected cases.

All GIS manipulations and cartographic displays were performed in ArcGIS 9.0 software.

## Results

### Study population

Approximately 1,800 trial participants were eligible for this sub-study. Men were passively recruited through information available at the clinic reception, posted fliers, and word of mouth. Between June 30, 2005 and March 13, 2006, we were able to recruit 1,040 men to the sub-study. This analysis is restricted to 649 of the 1040 men for whom residence coordinates were collected. For these 649 men, 185 (29%) were recruited at the baseline RCT visit, 218 (34%) at the 6-month, 140 (22%) at the 18-month visit, and 104 (14%) at the 24-month RCT visit. This sub-sample represents 23% of the RCT total sample (2,784 men). Participants were asked to locate the houses where they lived at the time of their enrollment into the trial. At the 6-month, 12-month, and 24-month RCT visit 80%, 73%, and 57% of the sub-study participants, respectively, indicated that they had not moved since enrolled into the trial.

Baseline characteristics of the 649 men are shown in Table 1. The majority of these men were single (95%) and unemployed (59%). Equal numbers of men had been randomized into circumcision and control groups. Engaging in insertive anal intercourse, paying or exchanging gifts for sex, and drinking alcohol with sex were reported infrequently (4%, 7%, 8%, and 11%, respectively). Condom use was measured by three different variables: in the past 6 months 66% reported ever using condoms; 33% used condoms more than half the time; and 53% used condoms the last time they had sex. Twenty-five percent reported having sexual dysfunction for a period of 2 weeks or longer in the past 6 months and 29% were diagnosed with one or more STIs at the baseline RCT visit. Men who joined this sub-study were less likely to be married (5% vs. 7% p = 0.01), more likely to have completed secondary school (61% vs. 55%, p = 0.001), more likely to report using condoms the last time they had sex (53% vs. 47%, p = 0.01), more likely to have no preference between wet and dry sex (×2 = 10.92, p = 0.03), more likely to initiate sex before the age of 15 (34% vs. 30%, p = 0.03), and less likely to report any of the sexual dysfunction items (p < 0.001 for all items) than those who did not enroll. There were no significant differences between the age of the participants (p = 0.49), randomization group (p = 0.88), importance of religion (p = 0.81), having sex the same day met a partner (p = 0.32), paying (p = .07) or giving gifts (p = 0.07) for sex, using condoms, whether always (p = 0.08) or half the time (p = 0.20), drinking alcohol last time had sex (p = 0.74), having anal sex with a woman (p = 0.67), and number of sex partners in the last 12 months (p = 0.25). There were no differences between the prevalent sexually transmitted infections at the baseline between those who enrolled in this sub-study and those who did not.

**Table 1:** Participants characteristics (n = 647)

	n	%
**Demographic characteristics**		

**Age group**		
18-20	337	52
21-24	310	48
**Trial randomization group**		
Circumcision	325	50
Control	322	50
**Marital status**		
Single	616	95
Married or cohabitating	29	5
Missing	2	0
**Employment**		
Salaried employee	72	11
Self-employed	189	29
No income	384	60
Missing	2	0
**Education**		
Primary	181	28
Any secondary	395	61
Post-secondary	71	11

**Sexual behaviors**		

**Sex the same day met a partner**		
Yes	207	32
No	438	68
Missing	2	0
**In past 6 months, paid money for sex^a^**		
Yes	46	8
No	530	92
Missing	2	0
**In past 6 months, exchanged gifts for sex^a^**		
Yes	52	9
No	524	91
Missing	2	0

**Condom use in the past 6 months^a^**		
Once or more	424	73
Never	152	26
Missing	2	0
**Condom use in the past 6 months^a^**		
More than half the time	213	37
Less than half the time	363	63
Missing	2	0
**Condom use last time had sex**		
Yes	339	53
No	306	47
Missing	2	0
**Had alcohol last time had sex**		
Yes	70	11
No	575	89
Missing	2	0
**Ever had anal sex with a woman**		
Yes	24	4
No	621	96
Missing	2	0
**Preference for dry vs. wet sex**		
Prefer dry sex	284	44
Prefer wet sex	242	37
No preference	74	11
Don't know	40	6
Refuse to answer	4	1
Missing	2	0
**Number of partners last 12 months**		
One	223	34
Two	182	28
Three or more	235	36
Missing	7	1
**Age at sexual debut**		
<15 years of age	209	32
≥ 15 years of age	349	54
Missing	25	4

**Sexually transmitted infections (STI) at baseline**		

**HSV2**		
Yes	159	25
No	464	72
Missing	24	4
**Syphilis**		
Yes	6	1
No	622	96
Missing	19	3

**Trichomonas vaginalis**		
Yes	6	1
No	631	98
Missing	10	1
**Neisseria gonorrhoeae**		
Yes	11	2
No	625	96
Missing	11	2
**Chlamydia trachomatis**		
Yes	28	4
No	607	94
Missing	12	2
**Any STI at baseline**		
Yes	186	29
No	433	67
Missing	28	4

**Sexual dysfunction for 2 or more weeks in the past six months^a^**		

**Lack of interest in sex**		
Yes	124	21
No	451	78
Missing	3	1
**Inability to come to a climax**		
Yes	7	1
No	568	98
Missing	3	1
**Came to a climax too quickly**		
Yes	50	9
No	523	90
Missing	5	1
**Pain during intercourse**		
Yes	22	4
No	553	96
Missing	3	1
**Sex not pleasurable**		
Yes	27	5
No	548	95
Missing	3	1
**Trouble achieving/maintaining erection**		
Yes	23	4
No	552	96
Missing	3	1
**Any dysfunction in the past 6 months**		
Yes	160	28
No	415	72
Missing	3	1

^a^Excludes 69 participants who were not sexually active in the last 6 months (N = 578)

### Distribution of high rate spatial clusters

**Figure 1:**
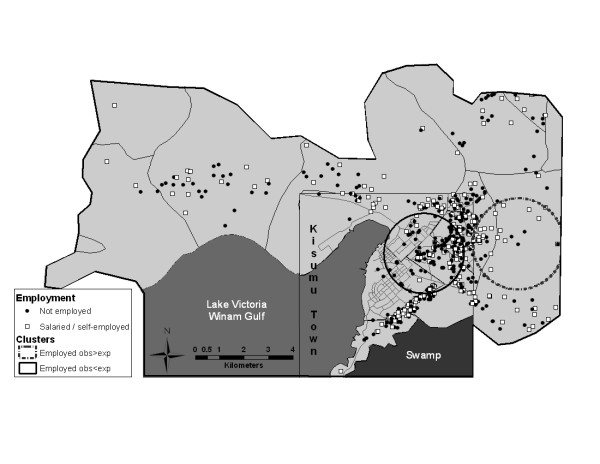
**Results of cluster analysis for the employment variable**.

**Figure 2:**
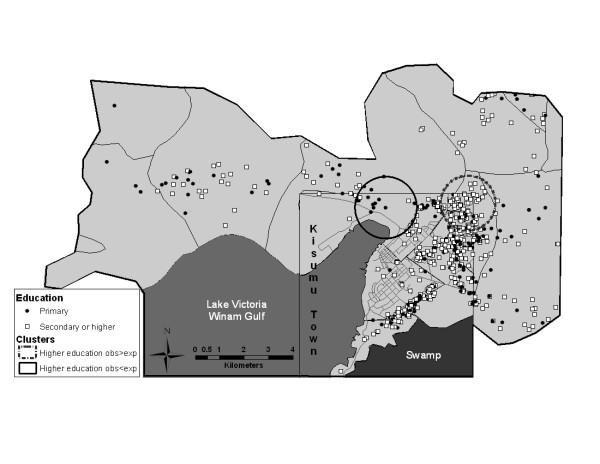
**Results of cluster analysis for the education variable**.

**Figure 3:**
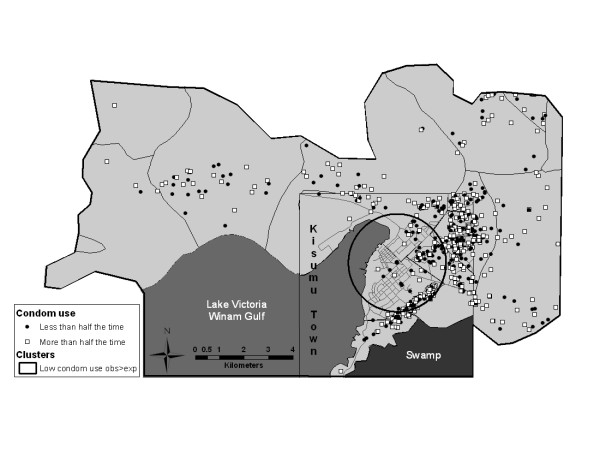
**Results of cluster analysis for the condom use variable (less than half the time compared to more than half the time)**.

Formal cluster analysis identified two statistically significant high rate clusters for education and employment variables (Table 2, Figures 1 and 2). The employment cluster was a small one (39 men, 31 of them employed and only 15.78 were expected to be employed, based on random distribution of cases) with a high cluster rate ratio of 1.96. This cluster was located in a rural area. The education cluster was larger, with a population of 95 participants, 88 of whom had secondary or higher level of education, compared to the expected number of 68.42. Most participants in the education cluster came from a middle-class neighborhood. The only statistically significant cluster identified in our sexual behavior variables was a cluster of men who used condoms less than half the time in the past 6 months (Table 2 and Figure 3). This cluster included 134 men, of which 73 reported using condoms less than half the time with 49.55 expected, resulting in a cluster rate ratio of 1.47. This geographically large cluster with a radius of 2 km covered a great part of central Kisumu, with a high concentration of participants residing in a sprawling low-income neighborhood.

**Table 2:** Results of cluster analysis: clusters with high rates (the observed number of cases is greater than the expected number of cases)

Case definition	Cluster Type	No. of cases in cluster	Expected No. of cases	LLR	Cluster Rate Ratio	P- value
**Sample Description**						

Age group: 21-24	Primary	11	5.27	8.20	2.09	0.23
Marital status: married or cohabitating	Primary	2	0.09	6.27	22.22	0.70
Importance of religion: quite or very important	Primary	38	30.25	8.97	1.26	0.10
Employment: salaried or self-employed	Secondary	31	15.78	13.20	1.96	**0.003**
Education: more than primary	Primary	88	68.42	14.49	1.29	**<0.001**
Education: more than primary	Secondary	124	104.44	9.31	1.19	0.07

**HIV risk behavior**						

Condom use in the past 6 months: once or more	Primary	36	27.24	8.13	1.32	0.16
Age at sexual debut: <15 years of age	Primary	8	2.69	8.83	2.97	0.13
Condom use in the past 6 months: less than half						
the time	Primary	73	49.55	11.17	1.47	**0.01**

**Sexual function in the last six months**						

For ≥ 2 weeks experienced lack of interest in sex	Primary	16	8.19	4.37	1.95	0.99
For ≥ 2 weeks was unable to come to a climax	Primary	2	0.073	5.35	27.40	0.43
For ≥ 2 weeks came to a climax too quickly	Primary	2	0.07	5.35	28.57	0.43
For ≥ 2 weeks experienced pain during intercourse	Primary	3	0.27	5.35	11.11	0.70
For ≥ 2 weeks had trouble achieving or maintaining an erection	Primary	3	0.28	5.22	10.71	0.73
Any dysfunction in the past 6 months	Primary	6	1.67	7.76	3.59	0.28

**Sexually transmitted infections at baseline**						

HSV2	Primary	5	1.28	6.89	3.91	0.43
Syphilis	Primary	2	0.096	4.72	20.83	0.52
Trichomonas vaginalis	Primary	3	0.27	5.39	11.11	0.36
Chlamydia trachomatis	Primary	2	0.09	6.31	22.22	0.67
Any STI at baseline	Primary	5	1.50	6.06	3.33	0.82

No statistically significant high rate clusters were identified for other sexual behaviors, nor for sexual dysfunction, or STI diagnosis variables.

### Distribution of low rate spatial clusters

Cluster analysis identified two statistically significant low rate clusters for education and employment variables (Table 3, Figures 1 and 2). The cluster for employment included 171 men, 39 employed and 69.20 expected to be employed based on simulations. This cluster was located in the northeast with a concentration of participants in a lower-income neighborhood of Kisumu town. The education cluster included 17 men, with only two of them reporting a higher level of education, compared to 12.24 expected, and was again located in a lower-income neighborhood.

**Table 3:** Results of cluster analysis: clusters with low rates (the observed number of cases is less than the expected number of cases)

Case definition	Cluster Type	No. of cases in cluster	Expected No. of cases	LLR	Cluster Rate Ratio	P- value
**Sample Description**						

Employment: salaried or self-employed	Primary	39	69.2	15.86	0.56	**<0.001**
Education: more than primary	Secondary	2	12.24	14.02	0.16	**0.001**

**HIV risk behavior**						

In past 6 months, paid money for sex	Primary	9	20.44	6.81	0.44	0.36
In past 6 months, exchanged gifts for sex	Primary	0	5.15	5.69	0.00	0.65
Sex the same day met with a partner	Primary	0	5.78	7.09	0.00	0.44
Used condom last time had sex	Primary	13	25.75	7.41	0.50	0.39
Had alcohol last time had sex	Primary	0	5.10	6.62	0.00	0.75
Ever had anal sex with a woman	Primary	0	6.14	7.25	0.00	0.18

**Sexual function in the last six months**						

For ≥ 2 weeks did not find sex pleasurable	Primary	0	4.74	5.36	0.00	0.77

**Sexually transmitted infections at baseline**						

Neisseria gonorrhoeae	Primary	0	5.50	7.72	0.00	0.18

No statistically significant low rate clusters were identified for any of the sexual behaviors, sexual dysfunction, or STI diagnosis variables.

### Results of ordinal and multinomial models

The results of ordinal and multinomial cluster analysis for education, preference for dry or wet sex, and number of partners in the last 12 months are presented in Table 4. When treated as a categorical variable, the cluster analyses for education resulted in the identification of three clusters. Cluster #1 had high rates of post-secondary education (RR = 2.07), and low rates of primary (RR = 0.62) and secondary education (RR = 0.98). Cluster #2 automatically collapsed the two higher education levels, resulting in high rates of secondary and above education (RR = 1.29) and low rates of primary education (RR = 0.26). Cluster #3 identified high rates of primary education (RR = 3.30) and low rates of secondary and post-secondary education (RR = 0.13 and RR = 0.00, respectively). No statistically significant clustering was detected for preference of dry versus wet sex and number of partners in the last 12 months.

**Table 4:** Results of cluster analysis for ordinal and nominal variables

Case definition	Cluster Type	No. of cases in cluster	Expected No. of cases	LLR	Cluster Rate Ratio	P- value
**Education: Cluster #1**	Primary			14.72		**0.004**
Primary		26	41.96		0.62	
Any secondary		90	91.58		0.98	
Post-secondary		34	16.46		2.07	

**Education: Cluster #2**	Secondary			14.49		**0.005**
Primary		7	26.58		0.26	
Any secondary or post-secondary		88	68.42		1.29	

**Education: Cluster #3**	Secondary			12.53		**0.011**
Primary		12	3.64		3.30	
Any secondary		1	7.94		0.13	
Post-secondary		0	1.43		0.00	

**Preference for dry vs. wet sex**	Primary			10.04		0.36
Prefer wet sex		40	28.36		1.41	
Prefer dry sex		16	33.28		0.48	
No preference		11	8.67		1.27	
Don't know		8	4.69		1.71	

**Number of partners in the last 12 months**	Primary			8.81		0.27
One		5	13.24		0.38	
Two		7	10.81		0.65	
Three or more		26	13.95		1.86	

## Discussion

Several studies have assessed differences in sexual behaviors and HIV prevalence in rural and urban settings in Kenya. A national level study based on the Kenya Demographic and Health Survey (KDHS) in 2003 found rural residence a protective factor for HIV infection in men, but not for women [38]. These national results may not reflect the situation in Nyanza province, however, where the HIV prevalence is the highest in the country and the epidemiology of HIV is likely unique. Voeten *et al. *[20] conducted a study examining urban and rural distinction in Nyanza province, finding that rural female residents reported higher levels of risky behavior than female respondents residing in urban settings. No differences were observed in males. The relative lack of spatial clustering noted in our sample of young men support this finding.

However, while Voeten and colleagues carefully constructed their sample to include 15-29 year old men and women representative of the corresponding population of one urban (Kisumu township) and two rural (Bondo and Siaya districts) areas, the sexually active young men constituting our sample can not be considered representative of a specific population. It is difficult, with the limitations of our study sample, to draw any conclusions based on our findings or directly compare the results of the two studies.

Analytically our method of cluster detection differed from both studies noted above allowing for the detection and definition of clusters based on individual-level spatial characteristics rather than a static designation of "urban" and "rural" areas. This methodology establishes the extent of heterogeneity across an entire study area, including intra-urban and intra-rural spatial clustering, and defines geographically localized groups of participants by measured characteristics. Consequently, areas of higher concentration of risky behaviors or HIV/STI cases are identified independent of administrative boundaries or classifications, such as urban or rural designation, that are designed to serve as "proxy" measures for underlying geospatial characteristics. Individual-level spatial measurements enrich the spatial characterization of a sample and can provide valuable information for programmatic implementations.

For example, the education clustering found in our study provides understanding of the study sample's educational and spatial heterogeneity beyond answering the question of whether rural-residing and urban-residing respondents differed in their educational attainment. One can see that education is not uniformly distributed by residence, and further that areas of educational clustering, both with more and less education than expected, extend across previously described urban and rural classification. Considering employment along with education, one can visualize that while there may be some spatial linkage, it is weaker than one might expect, with employment falling more clearly within the known urban and rural divide. With the limitations in our study sample any conclusions or interventions based on these groupings are not warranted, however our results do highlight that geospatial analytic tools can be of value. This usefulness increases in settings where administrative structures are not clearly defined, long periods have passed since populations were grouped and defined, or where population data are simply not available.

Considering our findings for this non-representative at risk group, the general lack of clustering, other than condom use, over the geographical area of the study indicates that there is little to no differentiation in sexual risk behavior based on home location in our sample. The lack of clustering of prevalent STIs is likely an extension of this relative equality in high-risk behavior in this sample. The noted localized clustering by education, but again not by sexual behaviors or STIs, suggests that education may not significantly contribute to variations in risk behaviors and STIs in this geographic context. Conducting additional analysis (results not shown), we observed that while education was associated with a number of sexual behaviors among the 649 men in our sub-study, we found no association between educational attainment and sexual behaviors among the men living within our two education clusters. This may be a sign that spatial distribution significantly impacts the relationship between education, sexual behaviors and STI prevalence. Extending this, we can describe a group more likely to choose a home location based on factors other than sexual risk (i.e. socio-economic factors) and, once chosen, a home location that has little impact on individual risk behavior.

Appreciating the uniqueness of our sample, we do note a single, geographically large, area where men reported condom use distinctive from men outside the cluster. Condom use was measured by three different variables: 1) using condoms at last sexual encounter, 2) ever using condoms in the past 6 months, and 3) using condoms more or less than half the time in the past 6 months. Condom use was localized only for 3) using condoms more or less than half the time, a question designed to gauge general propensity toward condom use with less polarity than the other two measures. If our sample was more representative of a defined population of interest, this finding of low condom use might be of interest as the area identified is west and north of independent population centers with well established HIV/STI programs. A possible gap in services could be considered.

As discussed, there were several limitations to this study. Because the study was restricted to a convenience sample of healthy, HIV-negative, sexually active 18-24 years-old men participating in the male circumcision trial, the sample cannot be considered representative of the general male population of Kisumu district. In addition, because the trial targeted high-risk young men, we were unable to detect behavioral and STI clustering across or within any other group (e.g. women or those at lower risk). Small numbers of cases for each STI may be responsible for our inability to detect clustering based on prevalence. We were unable to perform a meaningful analysis of the homosexual behaviors due to the extremely small number of study participants reporting a history of having sexual relations with men. Spatial analysis was limited to participants' residences and did not include geographic sites of socialization. As with any study of sexual behaviors, the validity of the self-reported responses may be affected by social desirability and reporting biases. RCT interviewers were highly skilled and extensively trained on techniques that may help in limiting such information biases, such as establishing rapport with clients prior to conducting the interview, and ensuring privacy and confidentiality. Asking men who enrolled into this sub-study during one of the later follow up (i.e., 6, 12, 18 or 24 months) RCT visits about their residence at the time of their enrollment in the trial may result in recall bias. However, the majority of participants reported maintaining the same residence since trial enrollment. Lastly, Kisumu East district has a mainly urban population and a rural environment that is relatively densely populated and closely tied to the urban center. This was, naturally, reflected in our study sample and may have affected our ability to detect and define "rural" clusters. Nonetheless, this remains one of the first studies to evaluate the spatial distribution of STIs and behaviors on an individual rather than aggregate level.

In conclusion, collecting data on the geographic location of study participants' residences, or other important locations, may be useful in the careful characterization of a study sample. Geospatial analysis can be an important tool in the investigation of HIV/STI risk factors in specifically defined populations of interest and in areas where the underlying population dynamic is poorly understood. HIV and STI control programs in sub-Saharan Africa have mainly focused on urban areas, because of higher HIV prevalence [6-10], concentration of high transmission areas, and the relative ease in the implementation of interventions in densely populated sites [39]. The results of this study, and the mixed results in other spatial work in general population, suggest the possibility that sexual risk behaviors and STIs are evenly distributed throughout the Kisumu district. Therefore, a geographically independent or neutral approach, rather than interventions targeting specific locations may be preferable. Integration of fine resolution geospatial data capture into future research will allow the important addition of true geographically based analytic techniques to HIV/STI research. Study populations including both men and women of wider age ranges and of various levels of identified risk will allow assessment of the spatial distribution of STIs and risk behaviors in more general populations, and are central to the consideration of our results.

## Competing interests

The authors declare that they have no competing interests.

## Authors' contributions

NW, SM, KA, JNA, CP, and RCB jointly designed the study and chose the methods for evaluation. KOA was involved in spatial design and execution. NW carried out the analysis and wrote the first draft of the manuscript. All authors were involved in the preparation of the manuscript. All authors read and approved the final manuscript.
